# Application of the workload indicators of staffing need method to calculate the size of the medical staff at a maternity hospital in the state of Bahia, Brazil

**DOI:** 10.1186/s12960-021-00660-6

**Published:** 2022-01-28

**Authors:** Angélica Araújo de Menezes, Catharina Leite Matos Soares, Mario Roberto Dal Poz, Isabela Cardoso M. Pinto

**Affiliations:** 1 Bahia State Health Department, Salvador, Bahia Brazil; 2 Institute of Collective Health, Federal University of Bahia, Salvador, Bahia Brazil; 3 Institute of Social Medicine, University of the State of Rio de Janeiro, Rio de Janeiro, Rio de Janeiro Brazil

**Keywords:** Workload, Staffing, Workforce planning

## Abstract

**Background:**

Functioning health systems require a health workforce (HWF) that is qualified, available, equitably distributed, and accessible to the entire population as the basis for guaranteeing access to health. There is a global HWF crisis, manifested in Brazil by unequal distribution of healthcare personnel, particularly in rural areas, urban peripheries, and other hard-to-reach communities, posing a major obstacle to guaranteeing access to health systems and services. Based on the above, calculating the size and analyzing the workloads of the medical staff in the Obstetrics Department (OD) and Urgent Care Center (UCC) in a state maternity hospital is relevant for designing improvements in the work processes and future strategies for recruiting, selecting, and retaining these workers at the hospital, in turn favoring improvement in the quality of care for women and children at the state level. This scenario motivated the study’s design, in which the overall objective was to analyze the workload of staff physicians working in the Obstetrics Department and Urgent Care Center of a public maternity hospital in the state of Bahia, based on the WISN method.

**Methods:**

This was an exploratory-descriptive intervention study with a quantitative approach and qualitative elements, using the methodological stages recommended by the WISN to calculate and analyze the workload of obstetricians working in the OD and UCC in the maternity hospital.

**Results:**

The study found a deficit of 14 shift obstetricians at the hospital with a workload of 0.81. The study also found that the insufficient number of obstetricians at the hospital resulted from precarious hiring formats, idle medical positions, and poorly structured work processes and a shortage in the multidisciplinary staff.

**Conclusion:**

The research sought to contribute to the reduction of the gap in models and methodologies for the staffing of gynecologists and obstetricians in Bahia Maternity Hospitals, without covering the whole subject, but to demonstrate that the findings of the workload analysis and its validation could be useful in promoting and directing the design and implementation of interventions to improve the quality of the workload.

## Background

Access to health is one of the United Nations Sustainable Development Goals. However, evidence shows that at least half of the global population lacks access to essential health services. The principal obstacle lies in the structuring of health services systems, which requires, among other factors, a health workforce (HWF) that is qualified, available, equitably distributed, and accessible to the entire population [1–6].

Skilled workers for health services provision require high investments and are difficult to obtain and indispensable for the health system to function properly. Thus, national and local health systems administrators face a daily challenge of efficiently managing health teams, a critical element for the more just distribution of the workload, aimed at greater productivity [7].

According to the publication *World Health Statistics 2018*, there is a global HWF crisis that affects nearly every country of the world, including Brazil. A shortage of health personnel is seen in rural areas, urban peripheries, and other hard-to-reach communities, with inequalities in HWF distribution, posing a major obstacle to the guarantee of access to health services and systems [6, 8–10].

To overcome this obstacle, a technique/methodology to calculate staffing needs that is reliable and applicable to various regional realities is an essential tool for decision-making in planning the health workforce (HWF) [8].

The Workload Indicators of Staffing Need (WISN) method, proposed by the World Health Organization (WHO) for calculating the size of healthcare teams, has shown great potential for predicting medical staffing in maternity hospitals, since the evidence produced in Brazilian and international studies shows that application of the WISN method appears to be adequate for identifying the components of the workload and sizing the number of health workers in the various professional categories, even in countries with completely different levels of care and health contexts [8, 11, 12].

Namibia underwent the first nationwide application of the WISN method, culminating in the updating of workforce standards in health units and in the establishment of evidence for new standards. The method calculated physicians, nurses, pharmacists, and pharmaceutical assistants. The findings highlighted the shortage of health workers and inequities in their distribution [12]. The same method was used in India, in the Ganjam District of the state of Orissa, to calculate the number of health workers needed for maternal and child health (MCH) under the National Rural Health Mission. The method’s application established indicators and parameters that can be applied elsewhere in India, besides evidencing a shortage of health workers in MCH [13].

In Brazil, a study applied the WISN method to predict human resources in nursing in a Family Health Unit (USF in Portuguese). The authors observed a balance between the workload proposed by the method and the number of health workers actually available in the USF [9].

Scientific evidence indicates a possible association between density of healthcare workers and maternal mortality rate, infant mortality, and immunization rates. Likewise, evaluations of national health programs have found that HWF shortage is one of the greatest obstacles to the implementation of evidence-based interventions to improve maternal and child health. Thus, predicting the number of healthcare workers needed to meet users’ needs in maternal and child health is essential for the improvement of this population’s health indicators [14].

Given the scarcity of Brazilian studies to base the calculation of physicians in maternal and child health, the current article analyzed the workload of OB/GYN physicians working in the Obstetrics Department (OD) and Urgent Care Center (UCC) in a public maternity hospital in the state of Bahia, Brazil, based on the Workload Indicators of Staffing Need method.

## Methods

A quantitative exploratory-descriptive intervention study was performed with qualitative elements, using the methodological stages recommended by the WISN to calculate and analyze the workload of OB/GYN physicians working in the Obstetrics Department (OD) and Urgent Care Center (UCC) of a maternity hospital located in Salvador, the state capital of Bahia, Brazil, selected by convenience, where the criterion was belonging to the state’s own healthcare system with direct administration.

The study site is a medium-sized hospital in which patient care is associated with teaching and research, part of the “Stork Network” for maternal and neonatal care, accredited for treatment of women that are victims of violence and performing termination of pregnancy in cases authorized under Brazilian legislation. The hospital has 100 beds, distributed as follows: 70 in Obstetrics (24 for surgical obstetrics and 46 for rooming-in), six in Gynecology, five in Neonatology, ten in the Conventional Neonatal Intermediate Care Unit, four in Kangaroo Intermediate Neonatal Care, one in Clinical Medicine, and four in the Day Hospital. It also includes an Urgent Care Center for mothers and newborns and an Obstetrics Department with three pre-labor, labor, and postpartum beds, three operating rooms, and four beds in the Post-Anesthesia Recovery Center.

A field diary was used to record the qualitative elements, with a space for annotating free observations identified by place, date, and time with the beginning and end of the activities [15] on 11 consecutive days.

The following stages were used to conduct the study, according to the WISN method (Table 1):

**Table 1:** Stages in the application of the WISN method Source: Primary data collected by the authors, 2019

Stages	Description
1st stage—definition of the unit, sector, and professional category	Choice of unit and sector in which the study will be performed according to the adequate profile for the study, as well as the professional categories for which the staffing need will be calculated
2nd stage—calculation of available working time	Available working time was calculated by multiplying the weekly workload by the number of weeks, subtracting absences (vacation and holidays, sick leave, and training)
3rd stage—definition of components of workload	Consisted of defining the most important work activities on the medical schedule [8, 9] divided into: health activities—performed by all the members of a professional category, identifying the work’s specificity, and generally recorded; support activities—those that complement the health activities, performed by all members of a professional category and generally not recorded; additional activities—complement the health activities, performed by some members of a professional category and whose statistics are not regularly recorded
4th stage—establishment of work standards	Identification of average time for the workload components, based on the service standard, category allowance standard (CAS), and individual allowance standard (IAS)
5th stage—establishment of standard workloads	Amount of work in a health service component that a health worker can perform in 1 year
6th stage—calculation of allowance factors	In this stage, we calculated the number of workers needed to perform the support activities, based on the CAS and IAS, which were converted to a category allowance factor (CAF) and individual allowance factor (IAF)
7th stage—staffing need based on the method	We determined the staffing need, based on WISN, to cover the health activities and support activities, calculated as the staffing need for the health activities, multiplied by the CAF and added to the IAF
8th stage—application and interpretation of data by the WISN method	Based on the two indicators furnished by the method, namely, the difference between the number of workers available in the unit and the necessary number and the ratio between these two values, called the WISN index. The latter, when close to one (~ 1.0), represents equilibrium between the available staff and the staffing demand to conduct the health unit’s workload; when greater than one (> 1.0) it shows a surplus in relation to the workload, and when less than one (< 1.0) it indicates that the current number of workers is insufficient to deal with the health unit’s workload

The staff obstetricians’ available working time was based on a weekly workload of 12 h, 1.47 days of vacation, 2.17 days of holidays, 0.88 days of sick leave, and 2.17 days of continuing education, showing that each physician provided the service with 45.32 weeks a year, with 45.32 days worked, corresponding to 543.84 h.

The following stages were used to define the activities (3rd stage) comprising the care provided by obstetricians in the OD and UCC and their 7tive times (4th stage):

- Meetings for alignment between the maternity hospital’s administrators and staff;
- Recording of the number of obstetricians comprising the hospital shift staff;
- Evaluation of the statistical data for 2018, provided by the institution;
- Workshops for prior determination of activities;
- Creation of a data collection instrument for the maternity hospital, aimed at testing the method and validating it, as needed;
- Direct observation of the time spent in each activity in predetermined time intervals;
- Observation and evaluation of workload factors with potential impact (workers’ overload that may lead to physical or mental sick leave or jeopardize patient safety);
- Technical consensus and discussion of the results achieved with the workers involved to validate the collected data and identify strengths and weaknesses and possible process improvements;

The data were collected from September 16 to 27, 2019, during the daytime shift, starting when the physician reached the hospital until the shift handover, totaling a minimum of 12 h of follow-up from Monday to Saturday. No observations were performed on the nighttime or Sunday shifts, since none of the physicians working on these shifts agreed to be interviewed. The mean time spent by the physicians on the activities was measured with a work sampling technique, which consisted of direct, structured, non-participant observation of a sample of seven physicians during their 12-h work shifts, always recorded by the same researcher.

## Results

The interventions/activities observed in the data collection in the UCC initially focused on counting the time spent on examinations and reexaminations in care for 26 patients, with the procedures ranging from admission to discharge from the maternity hospital. The main emergencies identified in this process involved women with preeclampsia, sepsis, and suspected diagnosis of uterine cancer with active hemorrhaging.

In the OD, the researcher recorded the duration of activities performed during the stay of 44 patients, 20 of whom in normal labor, two of whose deliveries involved instrumentation, and 24 of whom who underwent cesarian delivery, three with serious complications. The researcher also estimated the procedures performed in 20 patients who came to the unit following abortion, 14 evolving with manual vacuum aspiration (MVA) and six with uterine curettage. Per day, four routine examinations were performed per patient in the pre-labor, labor, and postpartum beds, but not all of them were observed by the researcher.

The workshop and data collection and treatment were followed by the determination of a list of seven patient care activities in the OD and two in the UCC, three support activities, and four additional activities (Table 2), described below:

- Patient care activities in the Obstetrics Department:
  Care for normal delivery with instrumentation
  Care for normal delivery without instrumentation
  Care for cesarean delivery
  Manual vacuum aspiration (MVA)
  Uterine curettage
  Care for severe patients
  Routine examination of patients in pre-labor, labor, and postpartum beds
- Patient care in Urgent Care Center:
  Medical evaluation
  Medical reevaluation
- Support activities in the Obstetrics Department and Urgent Care Center
  Shift handover
  Supervision of interns and residents
  Break
- Additional activities in the Obstetrics Department and Urgent Care Center
  Administrative meetings
  Preparation of work schedules
  Replies to inspections/ombudsman’s reports;
  Participation in committees

All the activities included time with preparation and execution, recording on official documents and patient files, movement between departments, and other activities directly and indirectly involved in patient care. The setup times^1^ were, respectively, 45 min for pre-labor, labor, and postpartum beds and 67 min for operating rooms, which were added to the times involved in normal and cesarean deliveries, forming the total time in the above-mentioned activities.

**Table 2:** Obstetricians’ activities and standard times in patient care, support, and related activities in the UCC and OD in a maternity hospital in Bahia, Brazil Source: Primary data collected by the authors, 2019

Workload component	Activities in workload component	Time spent in the activity	Unit of measurement
Patient care activities in the Obstetrics Department	Care for normal deliveries without instrumentation	152	Minutes/activity
	Care for normal deliveries with instrumentation	190	Minutes/activity
	Care for cesarean deliveries	175	Minutes/activity
	Manual vacuum aspiration (MVA)	124	Minutes/activity
	Uterine curettage	138	Minutes/activity
	Routine examination of patients in pre-labor, labor, and postpartum beds	147	Minutes/activity
	Care for severe patients	196	Minutes/activity
Healthcare activities in Urgent Care Center	Examination	20	Minutes/activity
	Reexamination	7	Minutes/activity
Support activities in Obstetrics Department and Urgent Care Center	Shift handover	20	Minutes/activity/day
	Break	150	Minutes/activity/day
	Supervision of interns/residents	47	Minutes/activity/day
Additional activities in the Obstetrics Department and Urgent Care Center	Administrative meetings	384	Hours/year
	Preparation of work schedules	72	Hours/year
	Replies to inspections	84	Hours/year
	Participation in committees	16	Hours/year

After surveying the activities and the respective times spent on them, we identified the need for an additional 16.53 and 36.03 obstetricians, respectively, for the UCC and OD. The largest numbers of obstetricians were needed in the activities “examination of patients in the UCC” (14.07), “care for cesarean delivery” (13.11), and “care for normal delivery” (11.92). Calculation of the need for healthcare personnel proceeded to the number of workers for support and additional activities, which was 76 workers, resulting from multiplication of 52.56 by the 1.43 category allowance factor, plus 1.02 obstetricians from the individual allowance factor (Table 3).

**Table 3:** Activity patterns of on-call obstetricians Source: *WISN*/WHO, 2019

A. Need for Professionals for Health Activities					
Activities	Number per year	Activity pattern	Unit of measurement	Standard Workload	Calculated need
Examination in Urgent Care Center	22,956	20	Minutes/activity	1,632	14.07
Reexamination in Urgent Care Center	11,478	7	Minutes/activity	4,661	2.46
Care for normal deliveries without instrumentation	2553	152	Minutes/activity	215	11.89
Care for normal deliveries with instrumentation	6	190	Minutes/activity	172	0.03
Care for cesarean deliveries	1620	264	Minutes/activity	124	13.11
Manual vacuum aspiration (MVA)	287	124	Minutes/activity	263	1.09
Uterine curettage	503	138	Minutes/activity	236	2.13
Care for severe patients	1022	196	Minutes/activity	166	6.14
Routine examination of patients in pre-labor, labor, and postpartum beds	365	147	Minutes/activity	222	1.64
Total					52.56

B. Need for Professionals for support Activities			
Activities	Value	Unit of measurement	CAS
Shift handover	20	Minutes/activity/day	2.78
Break	150	Minutes/activity/day	20.83
Supervision of interns/residents	46	Minutes/activity/day	6.53
Total: 30.17			CAF: 1.43

C. Need for Professionals for Additional Activities				
Activities	Number	Value	Unit of measurement	Yearly IAS
Administrative meetings	1	384	Hours/year	384
Preparation of work schedules	1	72	Hours/year	72
Replies to inspections	1	84	Hours/year	84
Participation in committees	1	16	Hours/year	16
Total: 556				IAF:1.02

To allow comparison of the staffing need and the existing number of OB/GYN physicians, we converted the various medical employment contracts and workloads to 12 h a week (Table 4).

**Table 4:** Number of medical job positions hired and executed, converted to 12 h a week Source: Primary data collected by the authors, 2019

Employment modality	Workload	Number of job positions hired	Number of job positions hired, converted to 12 h/week	Number of job positions executed	Number of job positions executed, converted to 12 h/week
State government	06 h/week	–	–	–	–
	12 h/week	13	21	13	21
	24 h/week	4	–	–	–
	36 h/week	–	–	–	–
CLT 1	06 h/week	–	–	–	–
	12 h/week	–	43	–	23
	24 h/week	20	–	10	–
	36 h/week	1	–	1	–
CLT 2	06 h/week	5	–	5	–
	12 h/week	9	17.5	9	17.5
	24 h/week	3	–	3	–
	36 h/week	–	–	–	–
Federal government	06 h/week	1	–	1	–
	12 h/week	–	0.5	–	0.5
	24 h/week	–	–	–	–
	36 h/week	–	–	–	–
Total	–	56	82	46	62

*CLT* consolidated labor legislation

Finally, the study indicated a shortage of 14 staff obstetricians at the maternity hospital, with a workload pressure of 0.81. The study also found that the insufficient number of obstetricians at the hospital was the result of precarious hiring practices and thus idle medical job position, besides poorly structured work processes and staff shortages in other health professions (Table 5).

**Table 5:** Need for obstetricians and existing workload Source: *WISN*/WHO, 2019

Professional category	Current number	Calculated need	Difference in number of obstetricians	WISN ratio
Obstetricians	62	76	− 14.11	0.81

Due to the diversity of employment contracts with different monthly earnings, the study found that the maternity hospital had difficulty filling the job positions that offered the lowest pay. Thus, among the total of 20 positions with 24-h workweeks, 50% were idle. If these positions had been filled, there would have been 82 obstetricians with 12-h workweeks in the activities (Table 4), and there would have been no shortage of staff obstetricians, since the *WISN* index would have been close to 1.0 (Table 6).

**Table 6:** Comparison of obstetric staff with complete occupation of the job positions Source: *WISN*/WHO, 2019

Professional category	Current number	Calculated need	Difference in number of obstetricians	WISN ratio
Physicians	82	76	5.89	1.08

The study also showed changes in the service standards, since this is a teaching hospital, and during the activities, when interns and/or residents were present, it was necessary for the staff obstetricians to provide explanations on the care, answer questions, and monitor the performance of care, thus increasing the time spent in the respective work activity.

Another factor that impacted the medical staffing was the shortage of nurses and nurse technicians, with workload pressures of 0.74 and 0.62, respectively (Table 7). This meant that the OB-GYN physicians had to perform nursing activities, due to the shortage of nursing staff during their shifts.

**Table 7:** Nursing and laboratory staffing need and workload pressure Source: Primary data collected by the authors, 2019

Job	Existing staff	Need	Shortage	Workload pressure
Nurses	68	110	− 42.00	0.62
Nurse technicians	84	113	− 29.00	0.74
Pathology lab technicians	18	28	− 10.00	0.64

The study also found long setup times for procedures in the operating room and pre-labor, labor and post-partum beds. Based on the score used in the United States of America, which classifies the operating room set up in three categories: poor > 40 min, average − 25–40 min and high performance < 25 min, setup times in operating rooms and pre-labor rooms, work and post-partum beds in this Brazilian maternity hospital were fair to poor, or 67 and 45 min [17].

Importantly, setup time is part of the total length of activities in cesarean and normal deliveries. Thus, a reduction in these setup times to acceptable levels would result in a reduction in the time spent in these activities and thus in the need for medical staff to cover these activities. The following is a list of the main factors impacting this indicator and identified during the fieldwork:

- Lack of an established process for mobilizing the rooms’ cleaning service.
- Delay in the turnaround of laboratory test results, due to:
  Insufficient number of lab technicians for the collection, analysis, and processing of samples, evidenced by a workload pressure of 0.64 (Table 7).
  Lack of integration between the laboratory system and hospital bed management.
- Shortage of nurse technicians in the OD (Table 7).

## Discussion

This pioneering study has the potential to contribute to planning and decision-making in the administration of the Bahia State Health Department (SESAB) and the specific maternity hospital. Knowledge on the numbers of staff obstetricians that are needed and the main factors causing the shortage may provide backing for designing improvements in the work processes and future strategies for recruiting, selecting, and retaining these healthcare workers at the hospital. This is because calculation of the HWF as a management tool orients workforce planning in the Unified Health System (SUS) and contributes to the quality of care and sustainability of the health services system [18, 19].

The study revealed that the obstetricians’ work overload is quite low (0.81), so the workload could reach the ideal parameters (close to 1.0) if all the job positions were filled by the SESAB through a Foundation and were in use at the hospital. Half of the 20 job positions with 24-h workweeks were actually idle, since the physicians only agree to work for this company for a maximum of 12 h a week. This has resulted in the lack of utilization of half of the hired workload. If the total weekly workload were implemented, there would be no shortage of staff obstetricians.

While the multiplicity of employment modalities shows the effort by the SESAB and the maternity hospital’s administration to guarantee continuity of care for women and children, since it demonstrates the attempt to hire staff to conduct the care, it also clearly illustrates how even physicians are not “immune” to increasingly precarious and deregulated labor conditions, suffering the impacts from diverse job formats and salaries, generating dissatisfaction in those who earn lower pay and contributing to the discontinuity of care, since lower pay contracts have difficulty retaining physicians at the hospital.

A study along this line showed that healthcare workers often submit their own resignations to search for better-paid jobs, considering the growth in Brazil’s health sector jobs in recent years [2].

However, it would be very reductionist to attribute the difficulty in retaining physicians exclusively to unequal pay, since the scientific literature reports that the main factor leading professionals to leave an organization is their level of job dissatisfaction, which can be caused by any one of the many aspects comprising the work [20].

According to the study’s results, the staff shortage at the maternity hospital is not limited to physicians, but also includes nursing and laboratory staff, who showed a higher work overload compared to the obstetricians. This reflects the increasingly precarious work conditions, the result of restructuring of production that affects all workers, the correlation of forces between the health professions, and physicians’ higher social and political status, leading to the administration’s greater effort at covering the shortage of medical staff compared to the other professions [21–26].

Medical staffing needs are always met more quickly when compared to the other health professions, which can also be explained by the predominantly physician-centered model of care in hospital structures. In the private medical care model, the physician is the main agent of healthcare acts, while the other professions are left with supporting roles [27]. The arrangement in the models of care associated with the physician’s social and political visibility, resulting from the technical and social division of labor, contribute to greater attention to filling physician shortages compared to other professions in the multidisciplinary team. Knowing that public funds are finite and that the hegemonic profession’s earnings are higher than those of the other health professions, one can infer that the situation contributes to aggravating the shortage of nonmedical healthcare workers.

As a consequence of shortages in the multidisciplinary staff, when work activities normally performed by nonmedical personnel are not covered by them, it impacts the performance of medical work. The low number of obstetric nurses meant that physicians were performing more than 93% of the normal deliveries. Contradictorily, a study in a large public maternity hospital in Rio de Janeiro showed that 44.6% of the normal deliveries were performed by obstetric nurses [28].

The shortage of nurse technicians also contributed to both the delay in the replenishment of testing materials and equipment and a work backlog for the nursing staff, culminating in the routine performance of some elementary activities by medical interns and residents, such as accompanying patients from bedside to the operating rooms and fetching materials and medicines for the OD.

The shortage of lab technicians led to delaying the delivery of preoperative exams to hospitalized patients already in the operating room, as required by the safety protocol, delaying the initiation of surgery and, ultimately, increasing the setup time [29].

During the observation period, physicians reported dissatisfaction with the performance of tasks that should be performed by other professional categories. As a result, the increase in the number of medical jobs without alignment with the deployment and planning of the entire multi-professional team would not increase the production and quality expected in the hospital studied [30].

One can thus infer that solving the physician shortage involves, among other factors, solving the shortages of nurse technicians, lab technicians, and obstetric nurses, since patient care results from interaction between the various professions, based on their complementary knowledge and practices to deal with the complexity of health needs in individuals and populations [26]. Thus, the lack of staff will impact both the quality and the capacity to provide the care.

The low work overload for physicians, identified in the study, could have been even more favorable, based on efforts to improve the work processes that directly impact setup time. A study on this issue showed that the time expended in cleaning and preparing operating rooms is one of the variables determining gain or loss of operating theaters’ operational capacity. Improvements in this area could thus produce gains not only for the performance of cesareans, MVAs and uterine curettages, but also elective and urgent gynecological surgeries [31].

The study’s limitations include the reduced field observation time and the fact that the observations were done from Monday to Saturday in the daytime shifts, which may have influenced the recorded times. Even if one agrees by technical consensus on the parameters of the unobserved activities, there is no way to guarantee that the times would be the same if identified by observation. The multiplicity of unintegrated systems in the performance of some of the calculations for the indicators in Excel tables also represents a limitation, since it not only increases the work but also makes the data susceptible to errors.

## Conclusion

The study analyzed the activities of obstetricians and gynecologists and their allocation in relation to the complexity of procedures in one maternity hospital. The results achieved were then used to test the methodology for workload indicators for staffing needs. The use of the WISN method showed a lack of HWF, not only for physicians (0.81 workload) but also for nurses, nurse technicians and laboratory workers with workload pressure, respectively, 0.62, 0.74 e 0.64.

It is important to recognize the invisibility of many health professionals in the context of healthcare practices, where the focus is mostly to replace medical staff, to the disadvantage of other health professions.

The research sought to contribute to the reduction of the gap in models and methodologies for the staffing of gynecologists and obstetricians in Bahia Maternity Hospitals, without covering the whole subject, but to demonstrate that the findings of the workload analysis and its validation could be useful in promoting and directing the design and implementation of interventions to improve the quality of the workload.

Although we would recommend that the study should be extended to the same maternity hospital for a longer period of observation, including other occupational health categories, and also to other maternity hospitals with similar profiles, to enhance the management of the health workforce.

## Data Availability

Available upon request to corresponding author.

## Abbreviations

CAF: Category allowance factor
CAS: Category allowance standard
HWF: Health workforce
IAF: Individual allowance factor
IAS: Individual allowance standard
MCH: Maternal and child health
MVA: Manual vacuum aspiration
OB/GYN: Obstetrician/gynecologist
OD: Obstetrics department
SESAB (in Portuguese): Bahia State Health Department
SUS (in Portuguese): Unified health system
UCC: Urgent care center
USF (in Portuguese): Family Health Unit
WHO: World Health Organization
WISN: Workload indicators of staffing need

## Footnotes

1 The time spent cleaning and preparing the operating room and adequate management of intervals between one procedure and another are important factors for promoting better results in the operating room (known as setup time) [16].
